# Extraordinary Case of Vicarious Menstruation

**Published:** 1844-07

**Authors:** F. C. Stewart


*Extraordinary Case of Vicarious Menstruation.* By Dr. F. C. Stewart.—Margaret Campbell, the patient thus affected, has had her arm swollen to an enormous size at five separate and distinct epochs, and always after an attack of dysmenorrhœa. The swelling has invariably continued until the restoration of the catamenial discharge; immediately after which, it begins to subside, and continues gradually to diminish until the arm is restored to its normal condition. It is now seven years since she first was troubled with it, and she has been as long as two years without having it to recur. Almost every variety of emmenagogue treatment has been pursued, and generally with very undecided effect. The arm has now been swollen since the month of September [this was on Nov. 18], and is so large as to measure fifteen and a half inches round the elbow, where it is the largest; the fingers and hand are likewise much enlarged and disfigured. The general health of the girl is good, and she experiences but trifling pain in her arm. She states that she is married, but has never borne children. The first invasion of the accident is attributed to her having put her hand into very cold water immediately after using some that was hot. Having her menses at the time, this imprudence checked them, and in a short time afterward the arm began to swell. So serious was the malady supposed to be by the surgeons who first saw it, that they are said to have recommended amputation.

[Since the foregoing statement was made to the society, we learn from Dr. Stewart that he has succeeded in restoring the menstrual discharge, by means of two setons, the one placed in the thigh, and the other in the hypogastric region; and that the arm has again returned to almost its natural size.]—*Ibid.*

